# Ultrasound with needle guiding exploration as a real-time modality for exploration of air rifle bullet close to cervical spine: A case report

**DOI:** 10.1016/j.ijscr.2021.105730

**Published:** 2021-03-05

**Authors:** Eko Setiawan, Said Shofwan, Sumadi Lukman Anwar, Shafira Zahra Ovaditya, Rheza Rizaldy, Grady Janitra

**Affiliations:** a Department of Surgery, Medical Faculty, Sultan Agung Islamic University / Sultan Agung Islamic Hospital, Semarang, 50164, Indonesia; b Department of Anesthesiology, Medical Faculty, Sultan Agung Islamic University / Sultan Agung Islamic Hospital, Semarang, 50164, Indonesia; c Department of Surgery – Faculty of Medicine, Public Health and Nursing, Universitas Gadjah Mada / Dr Sardjito Hospital, Yogyakarta, 55281, Indonesia; d Department of Biomedical Science, Medical Faculty, Sultan Agung Islamic University / Sultan Agung Islamic Hospital, Semarang, 50164, Indonesia

**Keywords:** Air rifle bullet, Real-time imaging, Ultrasonography, Case report, Guiding exploration

## Abstract

**Introduction and importance:**

Surgical exploration of retained air rifle bullet at the neck region is challenging because it is difficult to find bullets during exploration and prevent damage to vital structures. A bedside real-time imaging technique is needed to determine the retained bullet's location to the surrounding structures and guiding exploration.

**Case presentation:**

A 19-year-old male patient was admitted to the emergency department with neck pain after being shot by an air rifle. The patient's vital signs were stable. A small entry wound to the right lateral side of the neck without exit wound was found without neurological symptoms. The cervical X-ray showed the bullet at the third cervical vertebrae level. Surgical exploration was performed with needle-guiding ultrasonography. The bullet is a one-centimeter anterior transverse process of the third cervical vertebra marked by a hyperechoic object. Exploration was done by tracing the needle. Postoperative neurological evaluation was conducted, and no abnormalities were found.

**Clinical discussion:**

Preoperative imaging modalities are crucial before the surgical exploration of a retained air rifle bullet. X-ray and CT-scan are imaging modalities that are often used as an initial assessment of retained foreign bodies. However, ultrasonography provides advantages over X-ray and CT scan to provide real-time imaging that supports the surgeon while performing surgical exploration. Ultrasonography with needle guiding exploration increases the precise location of a retained air rifle bullet.

**Conclusion:**

Ultrasonography was a simple and precise real-time imaging modality for identifying and guiding the exploration of a retained air rifle bullet in the neck area.

## Highlights

• Real-time imaging modalities are needed while performing surgical exploration of retained foreign body.
• Ultrasonography offered an ability to visualize retained foreign body in a real-time.
• Needle guiding ultrasonography is a technique that allows surgeon to extracts the retained foreign body precisely.
• The combination of a needle guided ultrasonography, and other imaging studies may increase the accuracy of exploration.

## 1 Background

Surgical exploration of air rifle bullet at the neck region with conventional methods is difficult to perform, leading to vital structures injury [1]. A bedside real-time imaging technique is needed to determine the precise anatomical location and guide planning incision briefly with minimal complication [2]. Computed Tomography (CT) scan is a gold standard imaging technique for gunshot wounds in the neck region [3]. However, this technique is not real-time imaging and not always available bedside in every hospital in developing countries. Meanwhile, in this case, ultrasound was used as a bedside and real-time exploration guiding. This case report has been reported based on the SCARE Criteria [4].

## 2 Case report

**Fig. 1:**
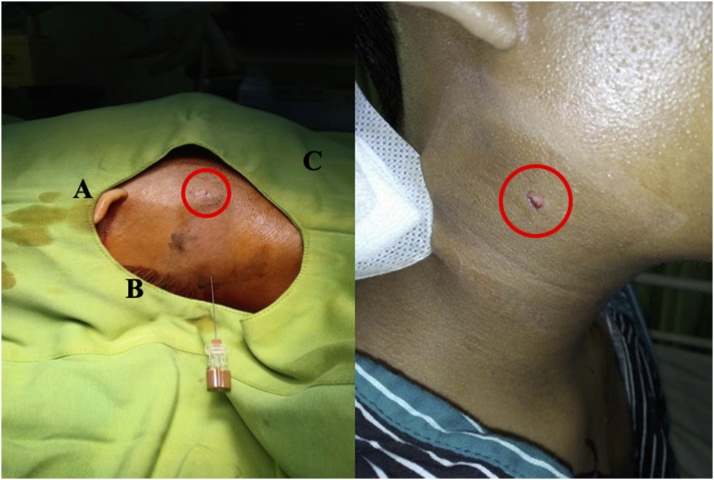
Entry wound (red circle) at the right lateral side of the neck region. A: Ear; B: Hair line; C: Angulus mandibular.

**Fig. 2:**
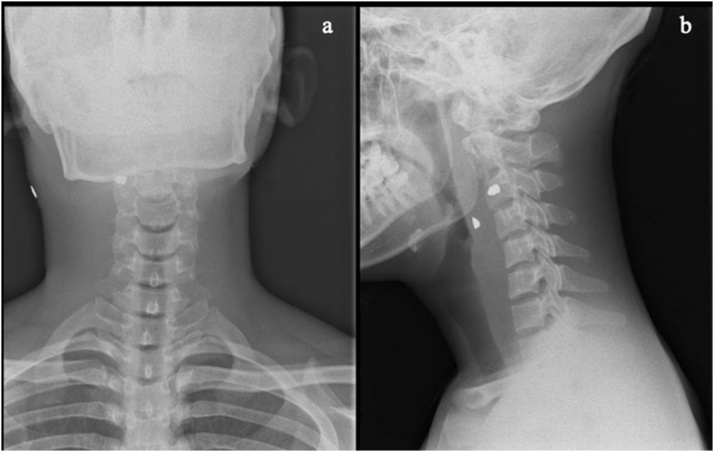
Cervical spine X-Ray. A: Anteroposterior view; B: Lateral view showed the bullet at the level of the third cervical vertebra.

A 19-year-old male patient was admitted to the emergency department with a pain complaint in the neck region after being shot by an air rifle. The patient's vital signs were stable. Wound characteristics were two millimeters diameter of entry wound on the right lateral side of the neck without an exit wound (Fig. 1). There were no injuries on other parts of the body. No neurological deficits were found. Cervical X-ray in anteroposterior and lateral view was taken; the metal foreign body in soft tissue five millimeters anterolateral from the third cervical vertebral body. The cervical spine was aligned properly, no listhesis or fracture were found (Fig. 2).

**Fig. 3:**
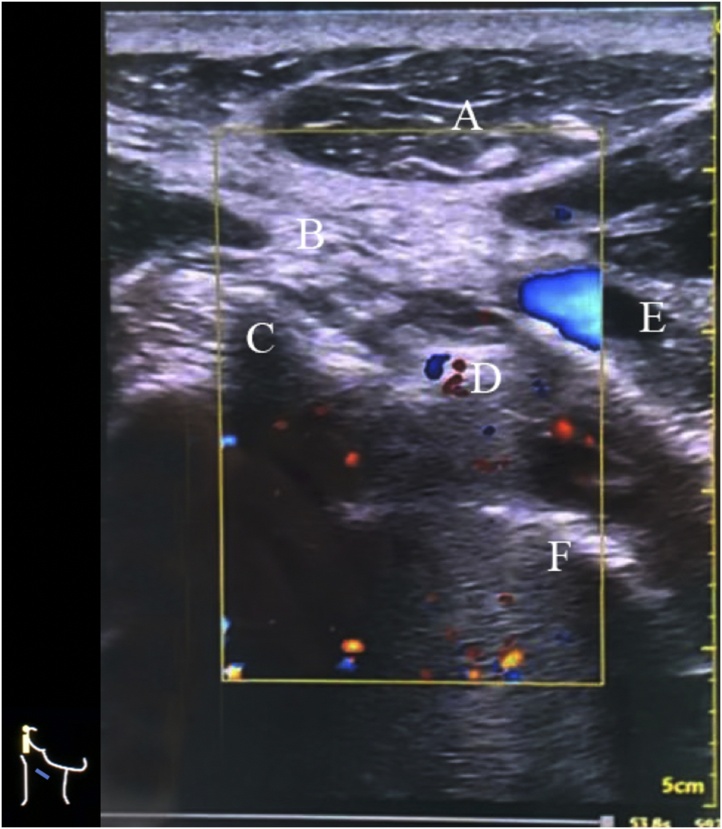
Ultrasound of the neck region. A: Sternocleidomastoid muscle; B: Needle’s track; C: Anterior tubercle; D: Air rifle bullet; E: Carotid artery; F: Vertebral body.

Surgical exploration was performed by a trauma surgeon with ultrasonography guiding by an expert. It was conducted guided by brightness-mode ultrasound. The entry wound was identified and matched with a cervical X-ray to estimate the bullet's position to the vertebral level and the depth from the skin. A foreign object was found in the form of a bullet approximately one centimeter anterior to the transverse process of the third cervical vertebra marked by a hyperechoic object with a comet tail sign. The surrounding anatomical structure was explored to estimate the direction of the marker needle to conserve vital structures (Fig. 3). The bullet was marked by the insertion of a spinal needle (25 Gauge) from the posterolateral side of the neck using an in-plane technique until the tip of the needle hit the bullet, marked by the tactile sensation felt in the operator's fingertip and the needle's position confirmed by out-plane technique. Exploration was done by tracing the tip of the needle. Postoperative neurological evaluation was conducted, and no abnormalities were found. The patient was satisfied with the result.

## 3 Discussion

Surgical exploration of an air rifle bullet in the neck requires preoperative imaging assessment to locate the bullet and estimate the possible organ damages. CT scanning is the gold-standard imaging in ballistic injury assessment and evaluation in hemodynamically stable patients [3]. However, this imaging not always available in a rural hospital and requires many preparations. Bedside X-ray is possible in hemodynamically unstable patients but cannot visualize soft tissue clearly. During surgical exploration, real-time imaging is needed because of the possibility of bullet migration. Unfortunately, the CT scan and X-ray cannot be performed bedside in real-time.

The primary goal of imaging in ballistic injuries is to specify the projectile's lane, assess injured tissues, calculate the damage's severity, and establish further studies. A conventional radiograph may identify ballistic fractures, as well as the location of bullet fragmentation. Even though it can show travel direction precisely, physicians are often challenging to differentiate between exit wounds and entry wounds [5]. CT scan is considered gold-standard imaging to visualize and identify ballistic injuries in stable patients. It has high sensitivity and specificity to define the wound tract and the involved viscera. CT scan may provide multi-plane reconstruction of the bullet and intra-parenchymal lesions. Unfortunately, not all hospitals in rural areas have CT scans as imaging for ballistic injuries. Furthermore, CT is no exact role in an unstable patient before surgical exploration [6,7].

The reports of ultrasonography regarding ballistic injuries appear to be limited. Ultrasound is a portable, quick and non-invasive imaging study method that offers a non-radiating modality to figure out gunshot wounds. It has an advantage over CT scans and conventional radiographs to allow real-time dynamic evaluations to confirm the lesions' accurate area before surgical exploration and describe the surrounding tissue environment [8,9]. However, ultrasound is very operator-dependent and difficult to perform quickly and precisely without experience. Speed is a crucial element in the emergency setting. The decision of the operator with the correct transducer and image characteristics such as depth, time/gain compensation, and brightness should be made rapidly to optimize diagnosis. Harcke et al. presented five cases of ballistic injuries and ultrasonography's role in locating bullets in the neck, abdomen, and extremities [10]. However, sonography was used as a diagnostic tool while presenting the utility of needle-guiding ultrasonography for surgical exploration. As of today, there have been no cases similar to this case.

Bedside ultrasonography is comparable with X-ray as a diagnostic tool in finding a retained foreign body, but ultrasonography is superior as a guiding device in surgical exploration for removing foreign body because it can help plan the surgery and to visualize surrounding anatomical structures. Sonographically guided foreign body exploration can also reduce the duration required from initial skin incision to remove the foreign body successfully [11,12]. Combining bedside ultrasound and plain radiography gives better sensitivity and specificity in detecting retained foreign bodies because plain radiography gives better visualization in detecting smaller radiopaque foreign bodies, while bedside ultrasound is better in visualizing larger and radiolucent foreign bodies [13]. One study showed successful foreign body removal in eight out of 11 cases [14].

We recommend a combination of imaging modalities to assess foreign bodies' exact location related to ballistic injuries. Needle-guided ultrasonography has been proven to be a useful adjunct to radiographic imaging for assessing foreign body location and surgical exploration management [15]. It can be used both as a diagnostic tool and guidance during surgical exploration. A tap or a tactile sensation of the foreign bodies felt in the operator's fingertip provides a more accurate and precise result [16]. Future research should be done to compare needle-guided ultrasonography and other radiographic imaging to explore the retained foreign body.

## 4 Conclusion

Needle-guiding ultrasonography is a promising imaging modality for surgical exploration of a deep retained foreign body because it is real-time imaging, portable, relatively quick, and non-invasive.

## Declaration of Competing Interest

All authors have declared that they have no potential competing interests.

## Sources of funding

We report no involvement of any sponsor or funding body for this study.

## Ethical approval

Ethical approval is not required at our Institution for case reports.

## Consent

Written informed consent was obtained from the patient for publication of this case report and accompanying images. A copy of the written consent is available for review by the Editor-in-Chief of this journal on request.

## Author contribution

ES conceptualized the first draft and finalized the manuscript. SZO, RR, and GJ wrote the manuscript. ES and SS involved in the surgery and care of the patient. SLA conceived the theoritical framework. All authors read and approved the final manuscript.

## Registration of research studies

Not applicable.

## Guarantor

Eko Setiawan.

## Availability of data and materials

The clinical and imaging data supporting this study's analysis and findings will be available from the corresponding author upon reasonable request.

## Provenance and peer review

Not commissioned, externally peer-reviewed.
